# Supplementary material for: A Comprehensive Assessment of Ultraviolet-Radiation-Induced Mutations in Flammulina filiformis Using Whole-Genome Resequencing
Source: J Fungi (Basel). 2024 Mar 20;10(3):228. doi: 10.3390/jof10030228 (PMC10971301; doi:10.3390/jof10030228)
Supplement: Supplementary file 1 [file jof-10-00228-s001.zip › Supplementary Material S8/KEGG annotation/out/64381550635650.os/KO/out_map/map03420.html]

KEGG PATHWAY: Nucleotide excision repair - Reference pathway


|  |  |
| --- | --- |
| **Nucleotide excision repair - Reference pathway** |  |

[
Pathway menu
| Organism menu
| Pathway entry
| Show description
| User data mapping
]

Nucleotide excision repair (NER) is a mechanism to recognize and repair bulky DNA damage caused by compounds, environmental carcinogens, and exposure to UV-light. In humans hereditary defects in the NER pathway are linked to at least three diseases: xeroderma pigmentosum (XP), Cockayne syndrome (CS), and trichothiodystrophy (TTD). The repair of damaged DNA involves at least 30 polypeptides within two different sub-pathways of NER known as transcription-coupled repair (TCR-NER) and global genome repair (GGR-NER). TCR refers to the expedited repair of lesions located in the actively transcribed strand of genes by RNA polymerase II (RNAP II). In GGR-NER the first step of damage recognition involves XPC-hHR23B complex together with XPE complex (in prokaryotes, uvrAB complex). The following steps of GGR-NER and TCR-NER are similar.

|  |  |
| --- | --- |
| Reference pathway | 100% |
